# Double overexpression of miR-19a and miR-20a in induced pluripotent stem cell-derived mesenchymal stem cells effectively preserves the left ventricular function in dilated cardiomyopathic rat

**DOI:** 10.1186/s13287-021-02440-4

**Published:** 2021-06-29

**Authors:** Jiunn-Jye Sheu, Han-Tan Chai, Pei-Hsun Sung, John Y. Chiang, Tien-Hung Huang, Pei-Lin Shao, Shun-Cheng Wu, Hon-Kan Yip

**Affiliations:** 1 Division of Thoracic and Cardiovascular Surgery, Department of Surgery, Kaohsiung Chang Gung Memorial Hospital and Chang Gung University College of Medicine, Kaohsiung, 83301 Taiwan; 2 Division of Cardiology, Department of Internal Medicine, Kaohsiung Chang Gung Memorial Hospital and Chang Gung University, College of Medicine, 123, Dapi Road, Niaosung Dist, Kaohsiung, 83301 Taiwan; 3 Center for Shockwave Medicine and Tissue Engineering, Kaohsiung Chang Gung Memorial Hospital, Kaohsiung, Taiwan; 4 Institute for Translational Research in Biomedicine, Kaohsiung Chang Gung Memorial Hospital, Kaohsiung, Taiwan; 5 Department of Computer Science and Engineering, National Sun Yat-Sen University, Kaohsiung, Taiwan; 6 Department of Healthcare Administration and Medical Informatics, Kaohsiung Medical University, Kaohsiung, Taiwan; 7 Department of Nursing, Asia University, Taichung, Taiwan; 8 Regenerative Medicine and Cell Therapy Research Center, Kaohsiung Medical University, No. 100, Shih-Chuan 1st Road, Kaohsiung, 807 Taiwan; 9 Orthopaedic Research Center, Kaohsiung Medical University, Kaohsiung, Taiwan; 10 Post-Baccalaureate Program in Nursing, Asia University, Taichung, Taiwan; 11 Department of Medical Research, China Medical University Hospital, China Medical University, Taichung, Taiwan; 12 Division of Cardiology, Department of Internal Medicine, Xiamen Chang Gung Hospital, Xiamen, Fujian China

**Keywords:** Dilated cardiomyopathy, Double overexpression of microRNAs, Oxidative stress, Inflammation, Mitochondrial damage

## Abstract

**Background:**

This study tested the hypothesis that double overexpression of miR-19a and miR-20a (dOex-mIRs) in human induced pluripotent stem cell (iPS)-derived mesenchymal stem cells (MSCs) effectively preserved left ventricular ejection fraction (LVEF) in dilated cardiomyopathy (DCM) (i.e., induced by doxorubicin) rat.

**Methods and results:**

In vitro study was categorized into groups G1 (iPS-MSC), G2 (iPS-MSC^dOex-mIRs^), G3 (iPS-MSC + H_2_O_2_/100uM), and G4 (iPS-MSC^dOex-mIRs^ + H_2_O_2_/100uM). The in vitro results showed the cell viability was significantly lower in G3 than in G1 and G2, and that was reversed in G4 but it showed no difference between G1/G2 at time points of 6 h/24 h/48 h, whereas the flow cytometry of intra-cellular/mitochondrial oxidative stress (DCFA/mitoSOX) and protein expressions of mitochondrial-damaged (cytosolic-cytochrome-C/DRP1/Cyclophilin-D), oxidative-stress (NOX-1/NOX2), apoptotic (cleaved-caspase-3/PARP), fibrotic (p-Smad3/TGF-ß), and autophagic (ratio of LC3B-II/LC3BI) biomarkers exhibited an opposite pattern of cell-proliferation rate (all *p*< 0.001). Adult-male SD rats (*n*=32) were equally divided into groups 1 (sham-operated control), 2 (DCM), 3 (DCM + iPS-MSCs/1.2 × 10^6^ cells/administered by post-28 day’s DCM induction), and 4 (DCM + iPS-MSC^dOex-mIRs^/1.2 × 10^6^ cells/administered by post-28 day’s DCM induction) and euthanized by day 60 after DCM induction. LV myocardium protein expressions of oxidative-stress signaling (p22-phox/NOX-1/NOX-2/ASK1/p-MMK4,7/p-JNK1,2/p-cJUN), upstream (TLR-4/MAL/MyD88/TRIF/TRAM/ TFRA6/IKK_α/ß_/NF-κB) and downstream (TNF-α/IL-1ß/MMP-9) inflammatory signalings, apoptotic (cleaved-PARP/mitochondrial-Bax), fibrotic (Smad3/TGF-ß), mitochondrial-damaged (cytosolic-cytochrome-C/DRP1/cyclophilin-D), and autophagic (beclin1/Atg5) biomarkers were highest in group 2, lowest in group 1 and significantly lower in group 4 than in group 3, whereas the LVEF exhibited an opposite pattern of oxidative stress (all *p*< 0.0001).

**Conclusion:**

iPS-MSC^dOex-mIRs^ therapy was superior to iPS-MSC therapy for preserving LV function in DCM rat.

**Supplementary Information:**

The online version contains supplementary material available at 10.1186/s13287-021-02440-4.

Assuredly, idiopathic dilated cardiomyopathy (DCM), a primary non-ischemic cardiac muscle disease, is clearly recognized as the consequence of systolic dysfunction and dilatation of chamber size of the left or both of ventricles [1, 2]. In fact, vast data have revealed that this disease accounts for approximately one-third of heart failure (HF) patients and is associated with adverse clinical outcomes and unacceptable high morbidity and mortality [3–7]. Abundant clinical trials and clinical observation studies have shown that HF, caused by different disease entity, is an important cause of morbidity and mortality in industrialized countries, with an estimation up to 5.7 million people affected in the USA [8, 9] and 30–50 million patients worldwide [10], and 5-year mortality reaching 50% [11].

Despite remarkable progress has been established in both advanced pharmacological and non-pharmacological modalities to treat DCM-related HF, the number of patients for hospitalization and deaths of HF patients has increased steadily [12–16]. The aforementioned issues [11–15] highlight that the treatment of HF caused by DCM is currently an unmet need, suggesting that to develop a safe and efficacious strategic management for DCM is of utmost importance and urgency to patients and physicians.

Inflammation is believed as a distinctive hallmark of HF [17, 18] in circulation and proinflammatory cytokines in the myocardium [17, 19–21]. This inflammatory response has been further identified in damaged myocardium during propagation of chronic HF to promote monocyte activation and the further production of cytokines, thus augmenting cardiac dysfunction [22], resulting in myocardial fibrosis and cardiac remodeling [23]. Additionally, studies have previously further displayed that an increased reactive oxygen species (ROS) plays a crucial role in the sudden death of these HF/DCM patients [24–26]. Furthermore, plentiful clinical and experimental studies have shown that mitochondrial dysfunction plays a principal role in the histopathological process of heart disease [27–29].

Recently, the utilization of human induced pluripotent stem cell-derived mesenchymal stem cells (iPSC-MSCs) has emerged as an innovative option for regenerative medicine [30, 31] and as a therapeutic alternative for various disease entities [32–34] mainly through suppressing the inflammation and generation of oxidative stress as well as the immunogenicity [35]. However, a full investigation of the impact of iPSC-MSCs on DCM has not been undertaken. Additionally, we have recently identified that the circulating levels of five anti-apoptotic micro-RNAs (i.e., miR-374a-5p, miR-19a-3p, miR-106b-5p, miR-26b-5p, and miR-20a-5p) were significantly lower in chronic kidney disease (CKD) patients than in healthy subjects [36]. These aforementioned issues raised the hypothesis that double overexpression of miR-19a and miR-20a (dOex-mIRs) in induced pluripotent stem cell (iPS)-derived mesenchymal stem cells (MSCs) (i.e., iPS-MSC^dOex-mIRs^) might effectively preserve heart function in DCM rat.

## Materials and methods

### Ethics

All animal procedures were approved by the Institute of Animal Care and Use Committee at Kaohsiung Chang Gung Memorial Hospital (Affidavit of Approval of Animal Use Protocol No. 2019061902) and performed in accordance with the Guide for the Care and Use of Laboratory Animals.

Animals were housed in an Association for Assessment and Accreditation of Laboratory Animal Care International (AAALAC; Frederick, MD, USA)-approved animal facility in our hospital with controlled temperature and light cycles (24 °C and 12/12 light cycle).

### Methodology of in vitro study of cell culturing for differentiation of human iPSC into mesenchymal stem cells (MSCs)

The procedure and protocol of human iPSC culture for differentiation into MSCs have been described in our previous study [35] and detailed information was illustrated in supplementary Figure 1. In details, at day 1, the human iPSCs (mTeSR™1; StemCell, #28315) were first washed by 5 mL PBS, followed by 2 mL Accutase (Gibco, #A1110501; Accutase: PBS = 1:1); the incubator reaction continued for 1 min. The 2 mL KO DMEM/F12 (Gibco, #12660012) was added and the cells were collected in 15 mL centrifuge tubes for 5-min duration of centrifuge (200×*g*). The cells were then cultured in a 10-cm dish for 24 h in mTeSR™1 culture medium.

By day 2, the cells (mTeSR™1) were collected and washed by 5 mL PBS. STEMdiff^TM^-ACF Mesenchymal Induction Medium (StemCell, #05241) was added to incubator culture and proceeded for 24 h. The STEMdiff^TM^-ACF Mesenchymal Induction Medium was exchanged once per day from days 1 to 3. This procedure was repeated on days 3 to 6. On days 7 to 21, the procedure was repeated but the culture medium was refreshed every 3 days.

### miR-19a-3p and miR-20a-5p were candidates for double overexpression in iPS-MSCs (iPS-MSC^dOex-mIRs^) and treatment of DCM in rodent

The procedure and protocol were based on our recent report [37]. We had identified that miR-19a-3p and miR-20a-5p were the two most suitable candidates among the five miRNAs (i.e., miR-374a-5p/miR-19a-3p/miR-106b-5p/miR-26b-5p/miR-20a-5p) to be overexpressed (i.e., transfection) for the purpose of treatment of chronic kidney disease + ischemia-reperfusion animals [37]. In detail, transfections of miR-19a-3p and miR-20a-5p mimics efficiently augmented the miRNA expressions and further decreased related gene expressions. Transfections of mimics (25 nM) were conducted with TransIT-X2 Dynamic Delivery System (Mirus), by following the manufacturer’s instruction. The iPS-MSCs were recognized > 80% confluence on the day of transfection. TransIT-X2 reagent was mixed with miRNA mimics for 25 min at room temperature. The miRNA mimics-containing complexes were further distributed into cells. Two days later, relevant expressions of miRNAs and genes were validated by the real-time qPCR assay.

### DCM induction in rodent by doxorubicin (Dox) and animal grouping

Pathogen-free, adult male Sprague-Dawley (SD) rats (*n*=32) weighing 320–350 g (Charles River Technology, BioLASCO Taiwan Co. Ltd., Taiwan) were utilized in this study. The procedure and protocol of Dox-induced rodent DCM model have been described in detail in our previous report [29]. In detail, the accumulated dose of 12.5 mg per kg at 4 separated time points within 20 days (i.e., once every 5 days) in each rat by intraperitoneal (IP) administration was applied in the present study.

Animals were equally categorized into group 1 (sham-control, i.e., by IP administration of 1.0 cc saline four times within 20 days, followed by opening chest wall only at day 28 after DCM induction), group 2 (DCM only), group 3 [DCM + iPS-MSCs/1.2 × 10^6^ cells/administered by day 28 after DCM induction), and group 4 (DCM + iPS-MSC^dOex-mIRs^/1.2 × 10^6^ cells/administered by day 28 after DCM induction). The dosage of MSCs in the present study was based on our previous studies [35, 38]. In the current study, the animals in each group were euthanized by day 60 and the heart specimen was harvested in each animal for individual study.

### Procedure and protocol for iPS-MSCs or iPS-MSC^dOex-mIRs^ implanted into the left ventricular (LV) by day 28 after DCM induction

In detail, all animals were anesthetized by inhalational 2.0% isoflurane and placed in a supine position on a warming pad at 37 °C. Under sterile conditions, the heart was exposed by opening the chest wall via a left thoracotomy after intubation with animal ventilatory support. Rats receiving thoracotomy in groups 1 and 2. While iPS-MSCs were implanted into four different regions of the LV myocardium in group 3, the iPS-MSC^dOex-mIRs^ was implanted into four different regions of the LV myocardium in group 4. After the procedure, the thoracotomy wound was closed, and the animals were allowed to recover from anesthesia in a portable animal intensive care unit (ThermoCare**®**) for 24 h.

### Determinant LVEF by utilizing the transthoracic echocardiography

Transthoracic echocardiography was performed in each group prior to and on days 28 and 60 after DCM induction. The procedure was performed by an animal cardiologist blinded to this experimental design using an ultrasound machine (Vevo 2100, Visualsonics). M-mode standard two-dimensional (2D) left parasternal-long axis echocardiographic examinations were conducted. Left ventricular (LV) internal dimensions [end-systolic diameter (ESD) and end-diastolic diameter (EDD)] were measured at the mitral valve level of the left ventricle, according to the leading-edge method of American Society of Echocardiography, by using at least three consecutive cardiac cycles. The left ventricular ejection fraction (LVEF) was calculated as follows: LVEF (%) = [(LVEDD^3^-LVEDS^3^)/LVEDD^3^] × 100%.

### Western blot analysis of LV myocardium

The procedure and protocol have been described in detail in our previous reports [35–39]. Equal amounts (30 μg) of LV myocardial protein extracts were separated by 8-12% SDS-PAGE. After electrophoresis, the separated proteins were transferred onto a polyvinylidene difiuoride (PVDF) membrane (Amersham Biosciences, Amersham, UK). Nonspecific sites were blocked by incubation of the membrane in blocking buffer [5% nonfat dry milk in T-TBS (TBS containing 0.05%Tween 20)] at room temperature for 1 h. Primary antibodies against tumor necrosis factor (TNF)-α (1: 1000, Cell Signaling), nuclear factor (NF)-κB (1:1000, Abcam), tumor necrosis factor receptor-associated factor 6 (TRAF6) (1:2000, Abcam), toll-like receptor (TLR)-4 (1:1000, Novus), myeloid differentiation primary response 88 (MyD88) (1:1000, Abcam), myelin and lymphocyte protein (Mal) (1:1000, Abcam), TRIF (1:1000, Abcam), translocating chain-associated membrane protein (TRAM) (1:1000, Thermo Fisher Scientific), TNF receptor associated factor 6 (TRAF6) (1:2000, Abcam), IKK-α (1:5000, Abcam), IKK-ß (1:1000, Cell Signaling), nuclear factor of kappa light polypeptide gene enhancer in B-cells inhibitor, alpha (IKB-α) (1:1000, Cell Signaling), apoptosis signal-regulating kinase 1 (ASK1) (1:1000, Abcam), phosphorylated mitogen-activated protein kinase 4 (p-MMK4) (1:1000, Cell Signaling), p-MMK7 (1:1000, Thermo Fisher Scientific), p-JNK1/2 (1:1000, Abcam), p-cJUN (1:1000, Abcam), Atg5 (1:1000, Cell Signaling), Beclin1 (1:1000, Cell Signaling), interleukin (IL)-1ß (1:1000, Cell Signaling), matrix metalloproteinase (MMP)-9 (1:2000, Abcam), NOX-1 (1:1500, Sigma-Aldrich), NOX-2 (1:1000, Sigma-Aldrich), cytosolic cytochrome C (1:2000, BD), cyclophilin-D (1:3000, Abcam), dynamin-related protein 1 (DRP1) (1:1000, Cell Signaling), LC3B-II (1:2000, Abcam), LC3B-I (1:2000, Abcam), mitochondrial Bax (1:1000, Abcam), cleaved caspase 3 (1:1000, Cell Signaling), cleaved Poly (ADP-ribose) polymerase (c-PARP) (1:1000, Cell Signaling), Smad3 (1:1000, Cell Signaling), and transforming growth factor (TGF)-ß (1:1000, Abcam) were used. Signals were detected with horseradish peroxidase (HRP)-conjugated goat anti-mouse, goat anti-rat, or goat anti-rabbit IgG.

Immunoreactive bands were visualized by enhanced chemiluminescence (ECL; Amersham Biosciences), which was then exposed to Biomax L film (Kodak). For quantification, ECL signals were digitized using Labwork software (UVP).

### Immunohistochemical (IHC) and immunofluorescent (IF) studies

The procedures and protocols for IHC and IF examinations were based on our previous reports [35–39]. Briefly, specimens of LV myocardium were utilized for IHC and IF staining, rehydrated paraffin sections were first treated with 3% H_2_O_2_ for 30 min and incubated with Immuno-Block reagent (BioSB, Santa Barbara, CA, USA) for 30 min at room temperature. Sections were then incubated with primary antibodies specifically against, 8-hydroxy-2′-deoxyguanosine (8-OHdG), γ-H2AX (1:1000, Abcam), and CD14 (1:200, Thermo Fisher), while sections incubated with the use of irrelevant antibodies served as controls. Three sections of heart specimens from each rat were analyzed. For quantification, three randomly selected HPFs (400× for IF studies) were analyzed in each section.

### Histopathological finding of myocardial fibrosis

The procedure and protocol were based on our previous studies [29]. In detail, hematoxylin and eosin and Masson’s trichrome staining were utilized for the identification of the LV fibrotic area. Three serial sections of LV myocardium in each animal were prepared at 4 μm thickness by Cryostat (Leica CM3050S). The integrated area (μm^2^) of fibrosis on each section was calculated using the Image Tool 3 (IT3) image analysis software (University of Texas, Health Science Center, San Antonio, UTHSCSA; Image Tool for Windows, Version 3.0, USA). Three randomly selected high-power fields (HPFs) (100×) were analyzed in each section. After determining the number of pixels in each fibrotic area per HPF, the numbers of pixels obtained from three HPFs were calculated. The procedure was repeated in two other sections of each animal. The mean pixel number per HPF for each animal was then analyzed by calculating all pixel numbers and dividing by 9. The mean integrated area (μm^2^) of fibrosis in LV myocardium per HPF was obtained using a conversion factor of 19.24 (since 1 μm^2^ represents 19.24 pixels).

### MTT assay, qPCR analysis, and flow cytometric analysis for identification of total cellular and mitochondrial oxidative stress and membrane potential of mitochondria in iPS-MSCs

For the purposes of in vitro study, the culturing cells were categorized into G1 (iPS-MSC), G2 (iPS-MSC^dOex-mIRs^), G3 (iPS-MSC + H_2_O_2_/100uM), and G4 (iPS-MSC^dOex-mIRs^ + H_2_O_2_/100uM), respectively. The cells were finally collected for the flow cytometric analysis for assessment of total cellular (i.e., by H_2_DCFDA test) and mitochondrial (i.e., by Mito-SOX assay) oxidative stress and membrane potential of mitochondria [i.e., Tetramethylrhodamine, Ethyl Ester, Perchlorate (TMRE assay)].

Additionally, the MTT assay was utilized in the present study to determine the cellular metabolic activity as an indicator of cell viability, proliferation, and cytotoxicity.

Furthermore, the cells were also collected after culturing for Western blot analysis. Finally, qPCR analysis was utilized to assess the success of overexpression of iPS-MSC^Oex-mIRs^.

### Procedure and protocol for measurement of reactive oxygen species (ROS)

The fluorescence and grayscale photos were captured by utilizing the DP controller 2.1.1.183 (Olympus). Grayscale photos for measuring the fluorescence intensity were processed by using Image J 1.37v (National Institutes of Health, USA). Nine grayscale photos from each slide were randomly acquired. As compared with the area of increased fluorescence intensity (IFI), the baseline fluorescence intensity (BFI) (arbitrary unit/400 × high-power field) was defined as the area in myocardium loaded without H_2_DCFDA. Six BFI areas were measured from each grayscale photo, from which 3 BFI areas were randomly chosen. The mean IFI and mean BFI were then calculated. The ratio of IFI to the BFI was determined as the relative fluorescence intensity.

The LV specimen were obtained, frozen rapidly in liquid nitrogen, and then stored at − 80 °C.

### Statistical analysis

Quantitative data are expressed as mean ± SD. Statistical analyses were performed using SAS statistical software for Windows Version 8.2 (SAS Institute, Cary, NC, USA). One-way ANOVA was conducted followed by Bonferroni multiple comparison post hoc test for comparing variables among groups. A probability value < 0.05 was considered statistically significant.

## Results

### The results of in vitro studies (Figs. 1, 2, 3, and 4)

**Fig. 1:**
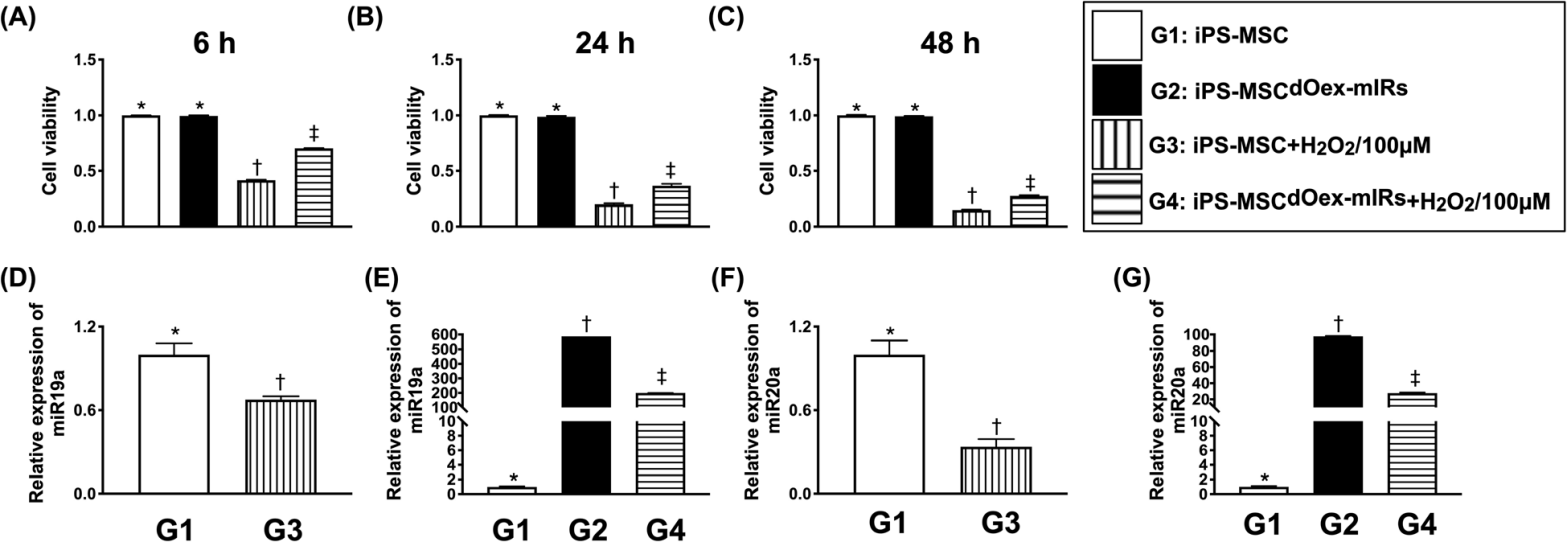
MTT assay for evaluating the impact of double overexpression of microRNAs on against the oxidative stress damage and qPCR. **A** By 6 h after cell culturing, the result of MTT assay, * vs. other groups with different symbols (†, ‡), *p*< 0.0001. **B** By 24 h after cell culturing, the result of MTT assay, * vs. other groups with different symbols (†, ‡), *p*< 0.0001. **C** By 48 h after cell culturing, the result of MTT assay, * vs. other groups with different symbols (†, ‡), *p*< 0.0001. *n*=8 for each group. G1= iPS-MSC; G2 = iPS-MSC^dOex-mIRs^; G3 = iPS-MSC + H_2_O_2_/100uM; G4 = iPS-MSC^dOex-mIRs^ + H_2_O_2_/100uM. **D** The qPCR analytical result of relative expression of miR-19a-3p in iPS-MSC with and without H_2_O_2_ treatment, * vs. †, *p*< 0.01. **E** The qPCR analytical result of relative expression of miR-19a-3p in iPS-MSC^dOex-mIRs^ with and without H_2_O_2_ treatment, * vs. other groups with different symbols (†, ‡), *p*< 0.0001. **F** The qPCR analytical result of relative expression of miR-20a-5p in iPS-MSC with and without H_2_O_2_ treatment, * vs. †, *p*< 0.01. **G** The qPCR analytical result of relative expression of miR-20a-5p in iPS-MSC^dOex-mIRs^ with and without H_2_O_2_ treatment, * vs. other groups with different symbols (†, ‡), *p*< 0.0001. *n*=4 for each group. dOex-mIRs = overexpression of double microRNAs (i.e., miR-19a-3p and miR-20a-5p). All statistical analyses were performed by one-way ANOVA, followed by Bonferroni multiple comparison post hoc test. Symbols (*, †, ‡) indicate significance (at 0.05 level)

To elucidate the cellular viability, the MTT assay was utilized. The result showed that this parameter was significantly higher in G1 (iPS-MSC) and G2 (iPS-MSC^dOex-mIRs^) than in G3 (iPS-MSC + H_2_O_2_/100uM) and G4 (iPS-MSC^dOex-mIRs^ + H_2_O_2_/100uM), and significantly higher in G4 than in G3 (Fig. 1), suggesting that overexpression of double microRNAs (i.e., miR-19a-3p and miR-20a-5p) was more resistant to oxidative stress damage.

**Fig. 2:**
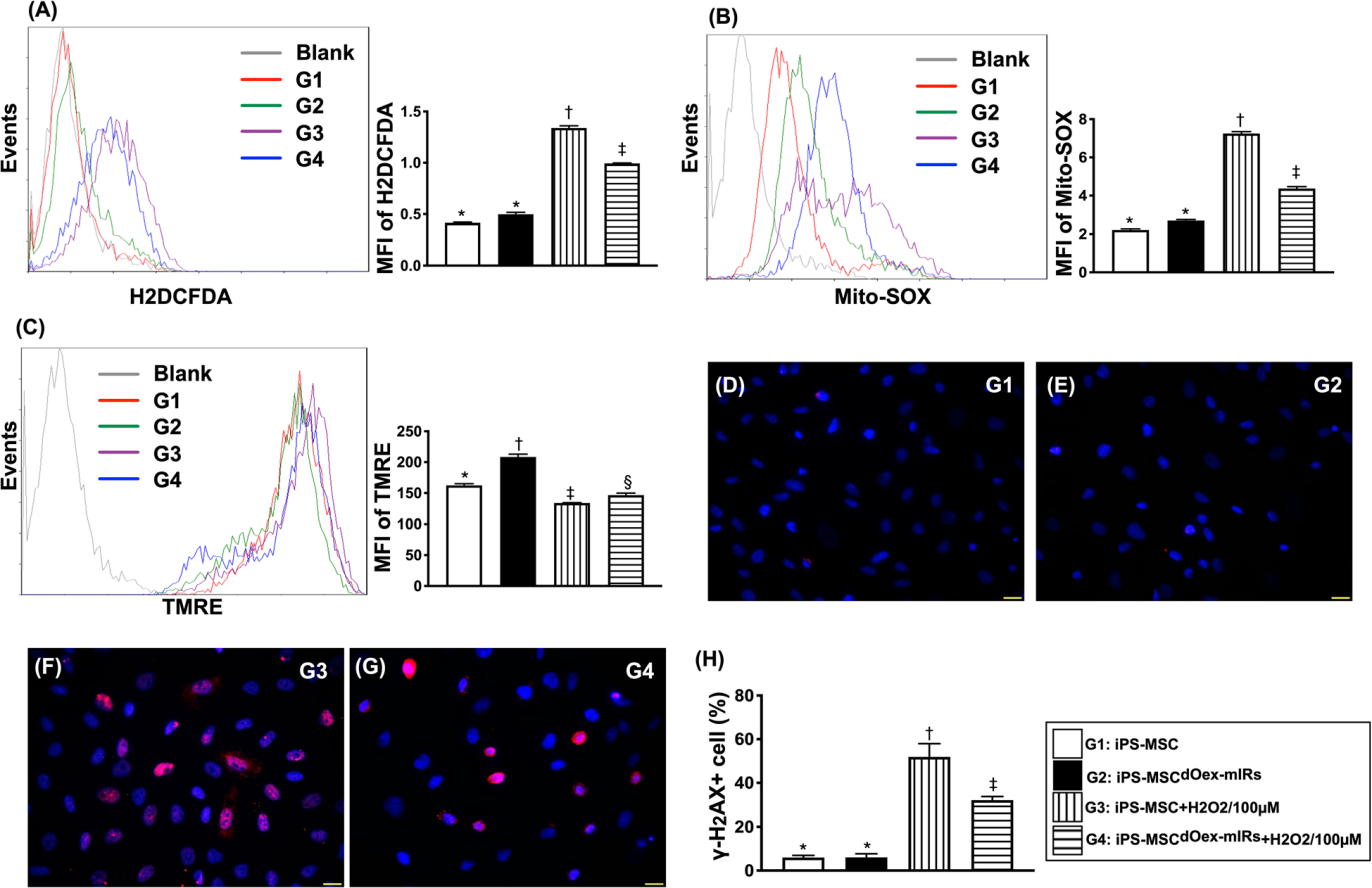
Flow cytometric analysis of oxidative stress and mitochondrial membrane potential in iPS-MSCs and iPS-MSC^dOex-mIRs^ and IF microscopic findings. **A** Fluorescent intensity of DCFDA (i.e., an indicator of total intracellular oxidative stress), * vs. other groups with different symbols (†, ‡), *p*< 0.0001. **B** Fluorescent intensity of Mito-SOX (i.e., indicator of mitochondrial oxidative stress), * vs. other groups with different symbols (†, ‡), *p*< 0.0001. **C** Fluorescent intensity of TMRE (i.e., an index mitochondrial membrane potential), * vs. other groups with different symbols (†, ‡, §), *p*< 0.0001. *n*=8 for each group. **D**–**G** Illustrating the immunofluorescent microscopic finding (400×) for identification of γ-H2AX+ cells (pink color). **H** Analytical result of positively stained γ-H2AX cells, * vs. other groups with different symbols (†, ‡), *p*< 0.001. *n*=4 for each group. Scale bars in the lower right corner represent 20 μm. All statistical analyses were performed by one-way ANOVA, followed by Bonferroni multiple comparison post hoc test. Symbols (*, †, ‡) indicate significance (at 0.05 level). MFI = mean fluorescent intensity; G1= iPS-MSC; G2 = iPS-MSC^dOex-mIRs^; G3 = iPS-MSC + H_2_O_2_/100uM; G4 = iPS-MSC^dOex-mIRs^ + H_2_O_2_/100uM

**Fig. 3:**
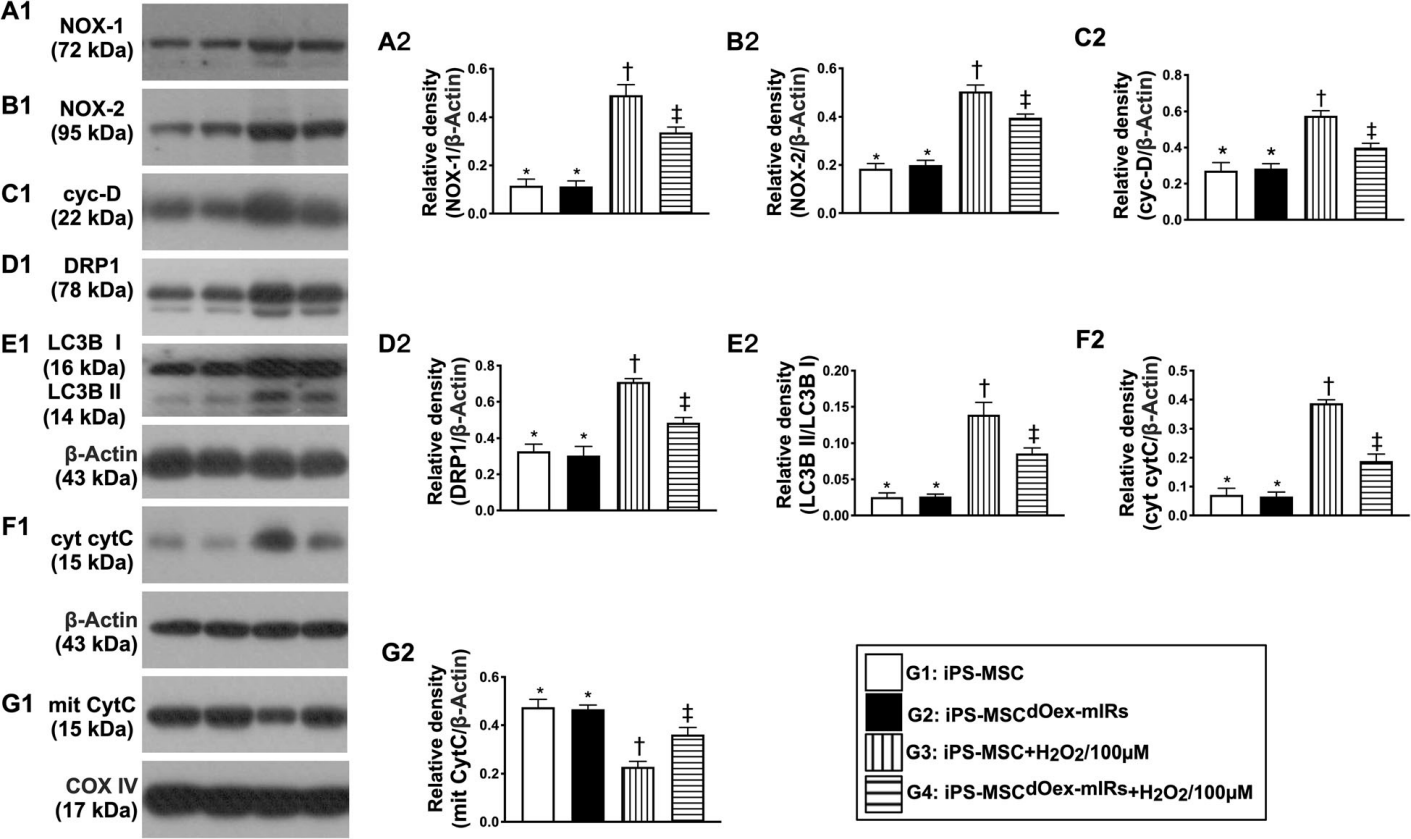
In vitro analysis of protein expressions of oxidative stress. **A1**, **A2** Protein expression of NOX-1, * vs. other groups with different symbols (†, ‡), *p*< 0.001. **B1**, **B2** Protein expression of NOX-2, * vs. other groups with different symbols (†, ‡), *p*< 0.001. **C1**, **C2** Protein expression of cyclophilin D (cyc-D), * vs. other groups with different symbols (†, ‡), *p*< 0.001. **D1**, **D**_**2**_ Protein expression of and dynamin-related protein 1 (DRP1), * vs. other groups with different symbols (†, ‡), *p*< 0.001. **E1**, **E2** Protein expression of ratio of LC3B-II to LC3B-I, * vs. other groups with different symbols (†, ‡), *p*< 0.001. **F1**, **F2** Protein expression of cytosolic cytochrome C (cyt-cytC), * vs. other groups with different symbols (†, ‡), *p*< 0.001 (Actin). **G1**, **G2** Protein expression of mitochondrial cytochrome C (mit-cytC), * vs. other groups with different symbols (†, ‡), *p*< 0.001. All statistical analyses were performed by one-way ANOVA, followed by Bonferroni multiple comparison post hoc test (*n*=3). Symbols (*, †, ‡) indicate significance (at 0.05 level). G1= iPS-MSC; G2 = iPS-MSC^dOex-mIRs^; G3 = iPS-MSC + H_2_O_2_/100uM; G4 = iPS-MSC^dOex-mIRs^ + H_2_O_2_/100uM

**Fig. 4:**
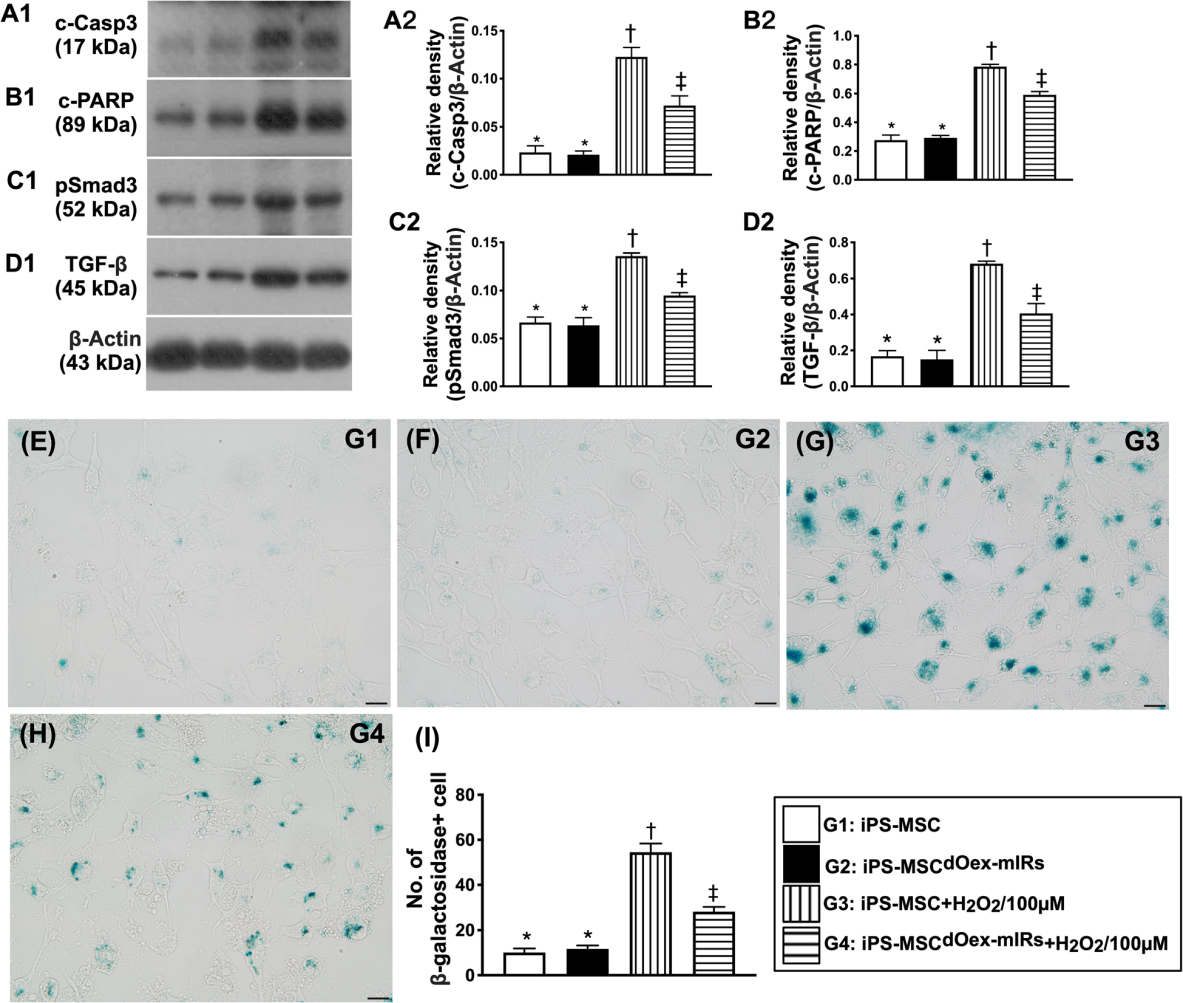
In vitro analysis of protein expressions of apoptosis and fibrosis cellular level of senescence. **A1**, **A2** Protein expression of cleaved caspase 3 (c-Casp3), * vs. other groups with different symbols (†, ‡), *p*< 0.001. **B1**, **B2** Protein expression of cleaved Poly (ADP-ribose) polymerase (c-PARP), * vs. other groups with different symbols (†, ‡), *p*< 0.001. **C1**, **C2** Protein expression of Smad3, * vs. other groups with different symbols (†, ‡), *p*< 0.001. **D1**, **D2** Transforming growth factor (TGF)-ß, * vs. other groups with different symbols (†, ‡), *p*< 0.001. **E**–**H** Illustrating the immunofluorescent microscopic finding (400×) for identification of positively-stained β-galactosidase cells (blue color). **I** Analytical result of anumber of β-galactosidase+ cells, * vs. other groups with different symbols (†, ‡), *p*< 0.001. Scale bars in the lower right corner represent 20 μm. All statistical analyses were performed by one-way ANOVA, followed by Bonferroni multiple comparison post hoc test (*n*=4). Symbols (*, †, ‡) indicate significance (at 0.05 level). G1= iPS-MSC); G2 = iPS-MSC^dOex-mIRs^; G3 = iPS-MSC + H_2_O_2_/100uM; G4 = iPS-MSC^dOex-mIRs^ + H_2_O_2_/100uM

Next, we assessed the capacity of microRNA transfection into iPS-MSCs by performing the relative quantitative qPCR. The result showed that relative miR-19a-3p and miR-20a-5p were lowest in G3, highest in G2, and significantly higher in G4 than in G1 (Fig. 1), suggesting that not only the miRs were successfully transfected into iPS-MSCs but also further proved that the dOex-mIRs still had a good capacity of transfection into iPS-MSC even in the situation of H_2_O_2_ treatment (i.e., oxidative stress).

Furthermore, to clarify whether the iPS-MSC^dOex-mIRs^ could offer a better ability to protect the cells against the increments of oxidative stress in cellular and mitochondrial levels as well as the active mitochondria of iPS-MSC^dOex-mIRs^ in the condition of oxidative stress, the flow cytometric analysis was performed. As we expected, the fluorescent intensity of DCFDA (i.e., an indicator of total intracellular oxidative stress) and Mito-SOX (i.e., an indicator of mitochondrial oxidative stress were highest in G3 than in G1 and G2, and those were significantly reversed in G4, whereas these parameters did not differ between G1 and G2 (Fig. 2). On the other hand, the TMRE, an index of membrane potential of mitochondria, was highest in G2, lowest in G3, and significantly lower in G4 than in G1 (Fig. 2).

To delineate whether the dOex-mIRs would protect the iPS-MSCs against the H_2_O_2_ induced DNA damage and cellular senescence, IF microscope was utilized. The results demonstrated the cellular expression of γ-H2AX (Fig. 2), an indicator of DNA damage, and the positively-stained β-galactosidase (Fig. 4) cells were significantly higher in G3 and G4 than in G1 and G2 and significantly higher in G3 than in G4, but they showed no difference between G1 and G2.

Moreover, by using the Western blot analysis, we investigated the impact of dOex-mIRs on ameliorating the oxidative-stress, mitochondria-damaged, apoptotic and autophagic biomarkers in iPS-MSCs. Again as our expected, the protein expressions of NOX-1 and NOX-2 (two indicators of oxidative stress), protein expressions of cytosolic cytochrome C, cyclophilin D, and DRP1 (three indices of mitochondrial-damaged parameters), protein expression of the ratio of LC3BI/LC3BII (an indicator of autophagy) (Fig. 3), protein expressions of cleaved caspase 3 and cleaved PARP (two indicators of apoptosis), and protein expressions of Smad3 and TGF-1ß (two indicators of fibrosis) (Fig. 4), were significantly increased in G3 than in G1 and G2, and those were significantly reversed in G4, but they did not differ between G1 and G2, whereas the protein expression of mitochondrial cytochrome (Fig. 3), an indicator of mitochondrial integrity, displayed an opposite pattern of oxidative stress among the four groups.

### The serial changes of LVEF and fluorescent intensity of oxidative stress in LV myocardium by day 60 after DCM induction (Fig. 5)

By day 0 (i.e., at baseline), the LVEF was similar among the group 1 (sham-operated control), group 2 (DCM only), group 3 [DCM + iPS-MSCs/1.2 × 10^6^), and 4 (DCM + iPS-MSC^dOex-mIRs^/1.2 × 10^6^ cells). However, by day 28 after DCM induction, the LVEF was significantly higher in group 1 than in groups 2 to 4, but it demonstrated no difference among groups 2 to 4. On the other hand, by day 60 after DCM induction, the LVEF was highest in group 1, lowest in group 2, and significantly higher in group 4 than in group 3, implicating that iPS-MSCs effectively and iPS-MSC^dOex-mIRs^ further effectively preserved heart function in setting of DCM.

**Fig. 5:**
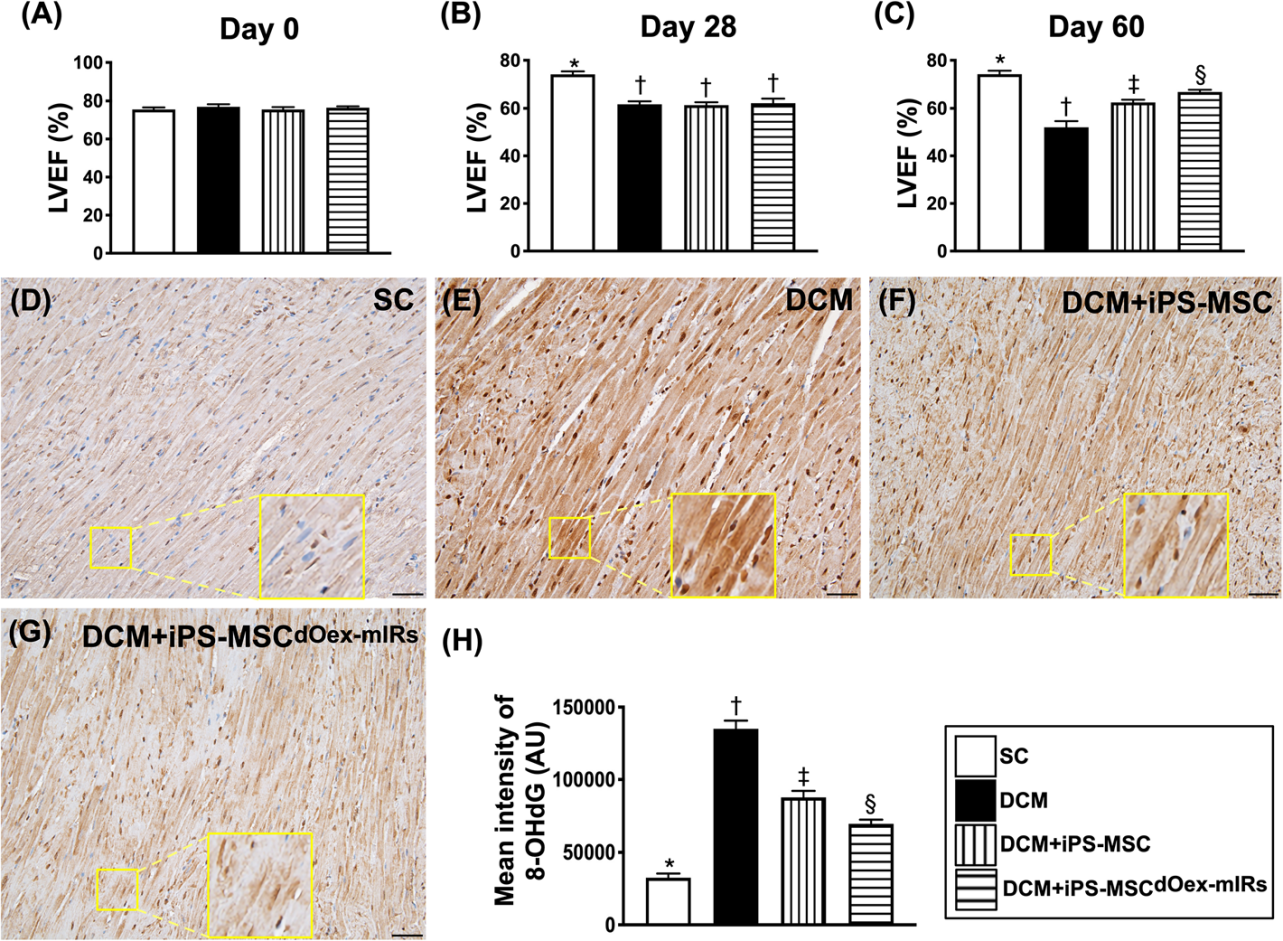
Serial changes of LVEF and fluorescent intensity of oxidative stress in LV myocardium by day 60 after DCM induction. **A** By day 0 prior to DCM induction, analytical result of the LVEF, *p*> 0.5. **B** By day 28 after DCM induction, * vs. †, *p*< 0.0001. **C** By day 60 after DCM induction, * vs. other groups with different symbols (†, ‡, §), *p*< 0.0001. *n*=8 for each group. **D**–**G** Illustrating the immunohistochemical microscopic finding (400×) for evaluation of fluorescent intensity [i.e., 8-hydroxy-2′-deoxyguanosine (8-OHdG) stain of myocardium specimen] of oxidative stress in LV myocardium (gray color). Note: the large square box was the result of magnification of small square box for more clearly to show the positively stained 8-OHdG in cardiomyocytes/troponin-I of LV myocardium. **H** Analytical result of fluorescent intensity of 8-OHdG staining in cardiomyocytes/troponin-I of LV myocardium, * vs. other groups with different symbols (†, ‡, §), *p*< 0.0001. Scale bars in the lower right corner represent 20 μm. *n*=4 for each group. All statistical analyses were performed by one-way ANOVA, followed by Bonferroni multiple comparison post hoc test. Symbols (*, †, ‡, §) indicate significance (at 0.05 level). LVEF = left ventricular ejection fraction; DCM = dilated cardiomyopathy; iPS-MSCs = inducible pluripotent stem cell-derived mesenchymal stem cells; dOex-mIRs = double overexpression of microRNAs

Additionally, the IF microscopic finding revealed that the fluorescent intensity of oxidative stress (i.e., by 8-OHdG stain) was highest in group 2, lowest in group 1, and significantly higher in group 3 than in group 4.

### Cellular expressions of DNA-damaged and inflammatory biomarkers in LV myocardium by day 60 after DCM induction (Fig. 6)

The IF microscopic finding demonstrated that the protein expressions of γ-H2AX+ cells, a DNA-damaged indicator, and CD14+ cells, an indicator of inflammation, were highest in group 2, lowest in group 1, and significantly higher in group 3 than in group 4.

**Fig. 6:**
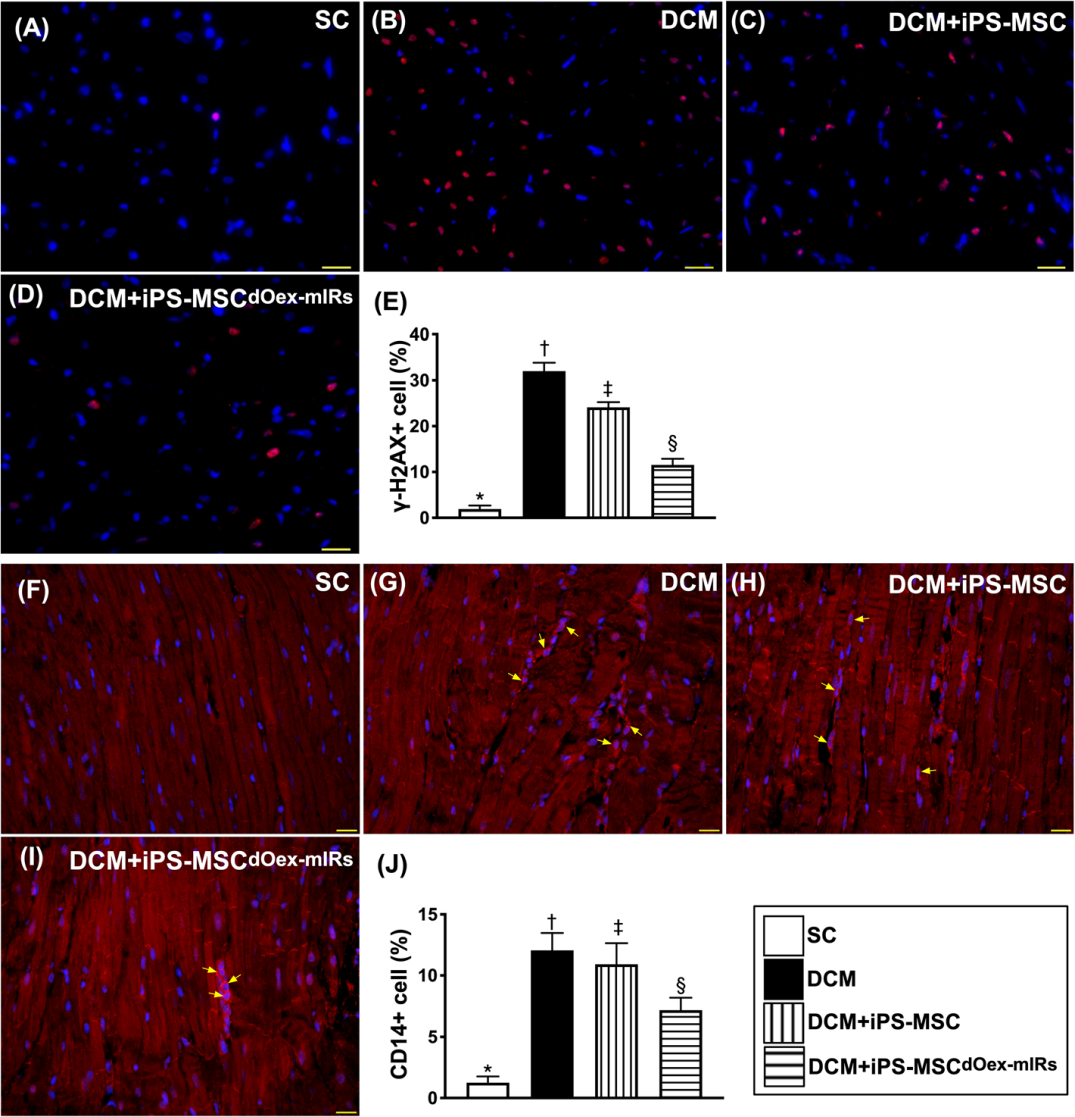
Cellular levels of DNA-damaged and inflammatory biomarkers in LV myocardium by day 60 after DCM induction. **A**–**D** Illustrating the immunofluorescent (IF) microscopic finding for identification the expressions of γ-H2AX+ cells (pink color). **E** Analytical result of a number of γ-H2AX+ cells, * vs. other groups with different symbols (†, ‡, §), *p*< 0.0001. **F**–**I** Showing the IF microscopic finding for identification of CD14+ cells (green color). **J** Analytical result of a number of CD14+ cells, * vs. other groups with different symbols (†, ‡, §), *p*< 0.0001. Scale bars in lower right corner represent 20 μm. All statistical analyses were performed by one-way ANOVA, followed by Bonferroni multiple comparison post hoc test (*n*=6 for each group). Symbols (*, †, ‡, §) indicate significance (at 0.05 level). LVEF = left ventricular ejection fraction; DCM = dilated cardiomyopathy; iPS-MSCs = inducible pluripotent stem cell-derived mesenchymal stem cells; dOex-mIRs = double overexpression of microRNAs

### Protein expressions of oxidative stress and mitochondrial damaged biomarkers in LV myocardium by day 60 after DCM induction (Figs. 7 and 8)

**Fig. 7:**
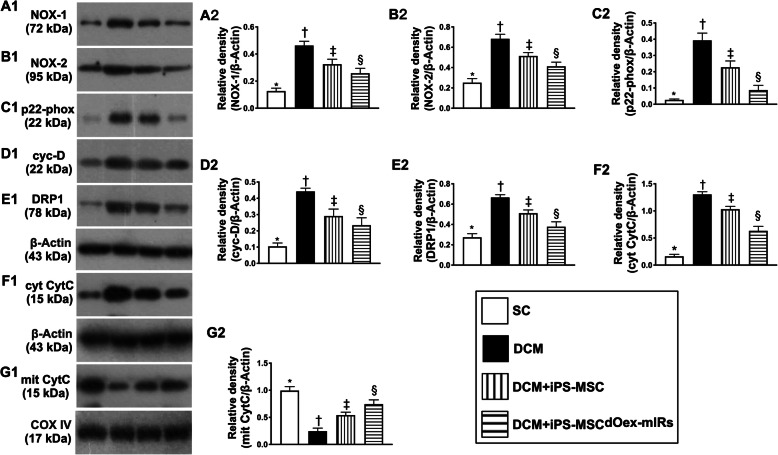
Protein expressions of oxidative stress and mitochondrial damaged biomarkers in LV myocardium by day 60 after DCM induction. **A1**, **A2** Protein expression of NOX-1, * vs. other groups with different symbols (†, ‡, §), *p*< 0.0001. **B1**, **B2** Protein expression of NXO-2, * vs. other groups with different symbols (†, ‡, §), *p*< 0.0001. **C1**, **C2** Protein expression of p22 phox, * vs. other groups with different symbols (†, ‡, §), *p*< 0.0001. **D1**, **D2** Protein expression of cyclophilin D (cyc-D), * vs. other groups with different symbols (†, ‡, §), *p*< 0.0001. **E1**, **E2** Protein expression of DRP1, * vs. other groups with different symbols (†, ‡, §), *p*< 0.0001. **F1**, **F2** Protein expression of cytosolic cytochrome C (cyt-CytC), * vs. other groups with different symbols (†, ‡, §), *p*< 0.0001. **G1**, **G2** Protein expression of mitochondrial cytochrome C (mit-CytC), * vs. other groups with different symbols (†, ‡, §), *p*< 0.0001. All statistical analyses were performed by one-way ANOVA, followed by Bonferroni multiple comparison post hoc test (*n*=6 for each group). Symbols (*, †, ‡, §) indicate significance (at 0.05 level). LVEF = left ventricular ejection fraction; DCM = dilated cardiomyopathy; iPS-MSCs = inducible pluripotent stem cell-derived mesenchymal stem cells; dOex-mIRs = double overexpression of microRNAs

**Fig. 8:**
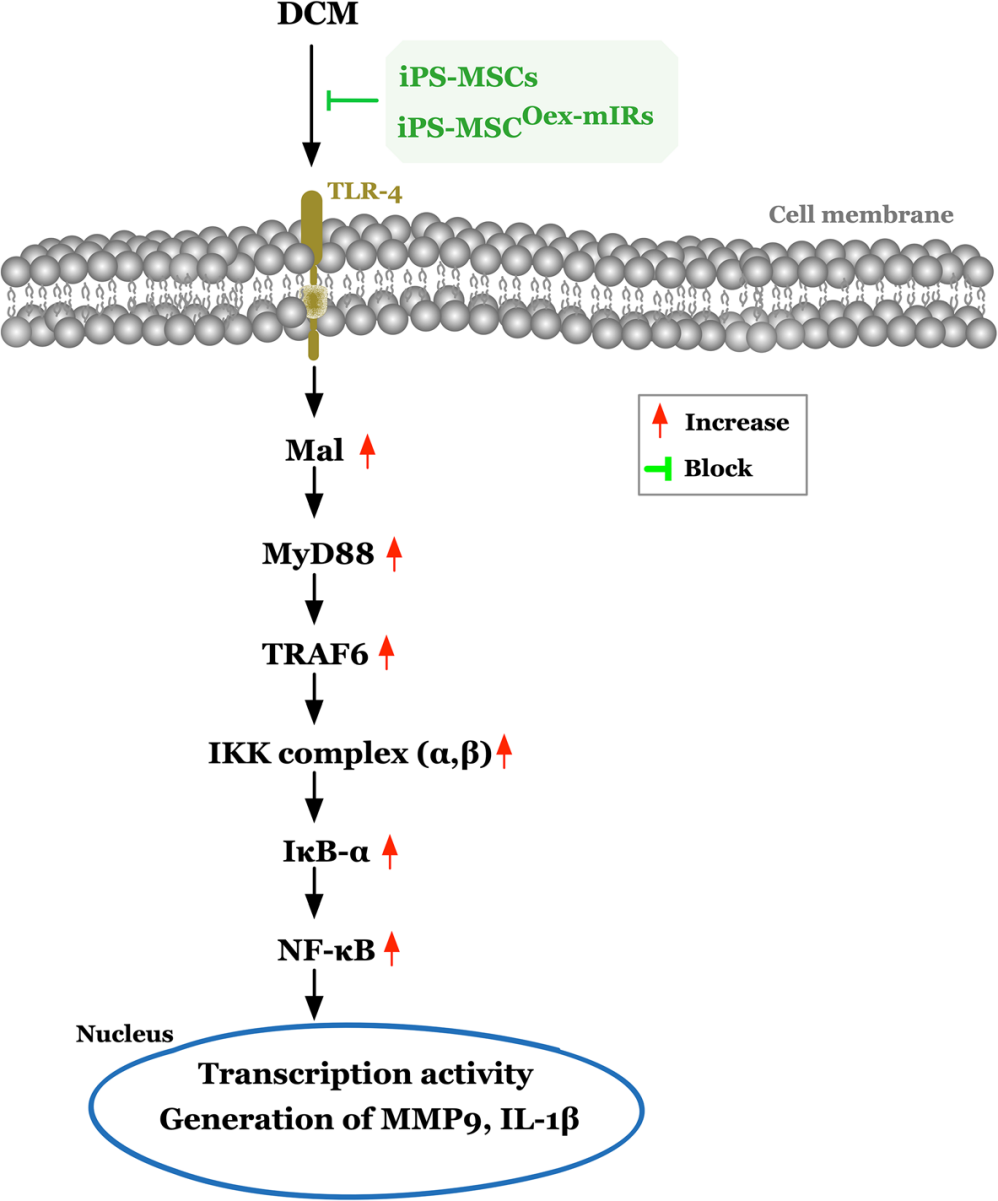
Schematically illustrated the underlying mechanisms of oxidative-stress and its downstream signaling that involved in the myocardial damage in DCM setting. DCM = dilated cardiomyopathy; ROS = reactive oxygen species; iPS-MSCs = inducible pluripotent stem cell-derived mesenchymal stem cells; dOex-mIRs = double overexpression of microRNAs

The protein expressions of NOX-1, NOX-2, and p22 phox, three indicators of oxidative stress, were significantly lower in group 1 than in groups 2 to 4, significantly lower in group 4 than in groups 2 and 3, and significantly lower in group 3 than in group 2 (Fig. 7). Additionally, the protein expressions of cytosolic cytochrome C, cyclophilin D, and DRP1, three indicators of mitochondrial damaged biomarkers, were lowest in group 1, highest in group 2, and significantly lower in group 4 than in group 3, whereas the protein expression of mitochondrial cytochrome C, an index of mitochondrial integrity, displayed an opposite pattern of oxidative stress among the four groups (Fig. 7). Our findings, in addition to delineating how the oxidative-stress signaling on damaging the myocardium (Fig. 8), suggested that iPS-MSCs effectively and iPS-MSC^dOex-mIRs^ more effectively protected the mitochondria through attenuating the upregulation of oxidative stress in DCM setting.

### Protein expressions of mitogen-activated protein kinase (MAPK) pathway, apoptosis and autophagic biomarkers in LV myocardium by day 60 after DCM induction (Fig. 9)

We also assessed the role of MAPK in DCM setting by Western blot. As we expected, the protein expressions of apoptosis signal-regulating kinase 1 (ASK1), p-MMK4, p-MMK7, p-JNK1/2, and p-cJUN, four members of MAP kinase family, were highest in group 2, lowest in group 1, and significantly higher in group 3 than in group 4. Additionally, the protein expressions of mitochondrial Bax, cleaved caspase 3, and cleaved PARP, three indicators of apoptosis, and the protein expressions of Atg5 and Beclin1, two indices of autophagic biomarkers, displayed an identical pattern of MAPK family among the four groups.

**Fig. 9:**
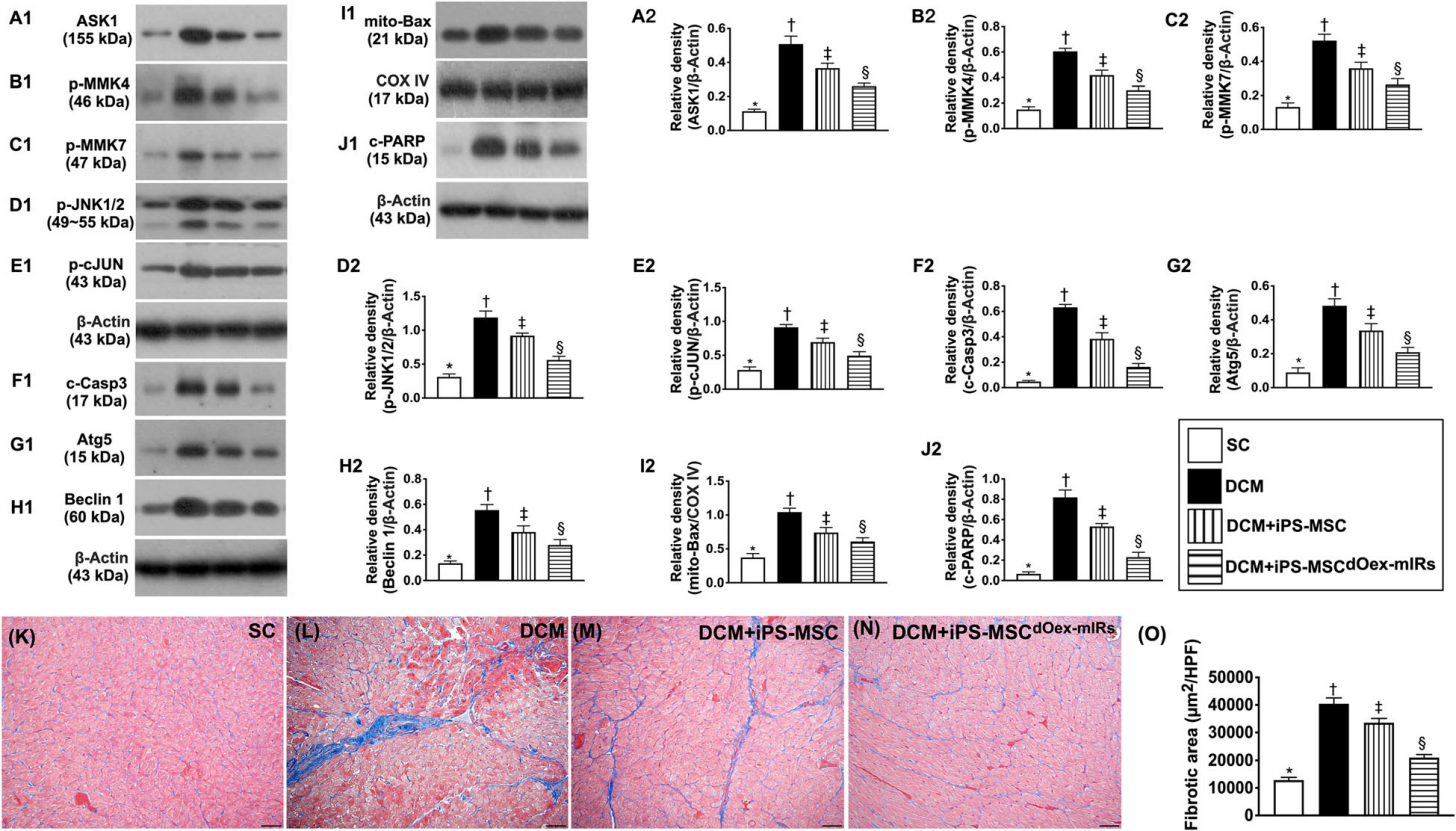
Protein expressions of mitogen-activated protein kinase (MAPK) pathway, apoptosis, autophagic and fibrotic biomarkers in LV myocardium by day 60 after DCM induction. **A1**, **A2** Protein expression of apoptosis signal-regulating kinase 1 (ASK1), * vs. other groups with different symbols (†, ‡, §), *p*< 0.0001. **B1**, **B2** Protein expression of phosphorylated mitogen-activated protein kinase 4 (p-MMK4), * vs. other groups with different symbols (†, ‡, §), *p*< 0.0001. **C1**, **C2** Protein expression of p-MMK7, * vs. other groups with different symbols (†, ‡, §), *p*< 0.0001. **D1**, **D2** Protein expression of p-JNK1/2, * vs. other groups with different symbols (†, ‡, §), *p*< 0.0001. **E1**, **E2** Protein expression of p-cJUN, * vs. other groups with different symbols (†, ‡, §), *p*< 0.0001. **F1**, **F2** Protein expression of cleaved caspase 3 (c-Casp3), * vs. other groups with different symbols (†, ‡, §), *p*< 0.0001. **G1**, **G2** Protein expression of Atg5, * vs. other groups with different symbols (†, ‡, §), *p*< 0.0001. **H1**, **H2** Protein expression of Beclin1, * vs. other groups with different symbols (†, ‡, §), *p*< 0.0001. **I1**, **I2** Protein expression of mitochondrial Bax (mito-Bax), vs. other groups with different symbols (†, ‡, §), *p*< 0.0001. **J1**, **J2** Protein expression of cleaved Poly (ADP-ribose) polymerase (c-PARP), * vs. other groups with different symbols (†, ‡, §), *p*< 0.0001. **K**–**N** Illustrating microscopic finding (200×) of the Masson’s trichrome stain for identification of fibrotic area in LV myocardium (blue color). **O** Analytical result of fibrotic area, * vs. other groups with different symbols (†, ‡, §), *p*< 0.0001. Scale bars in the lower right corner represent 50 μm. All statistical analyses were performed by one-way ANOVA, followed by Bonferroni multiple comparison post hoc test (*n*=6 for each group). Symbols (*, †, ‡, §) indicate significance (at 0.05 level). LVEF = left ventricular ejection fraction; DCM = dilated cardiomyopathy; iPS-MSCs = inducible pluripotent stem cell-derived mesenchymal stem cells; dOex-mIRs = double overexpression of microRNAs

### Protein expressions of upstream and downstream inflammatory signaling pathways in LV myocardium by day 60 after DCM induction (Figs. 10 and 11)

**Fig. 10:**
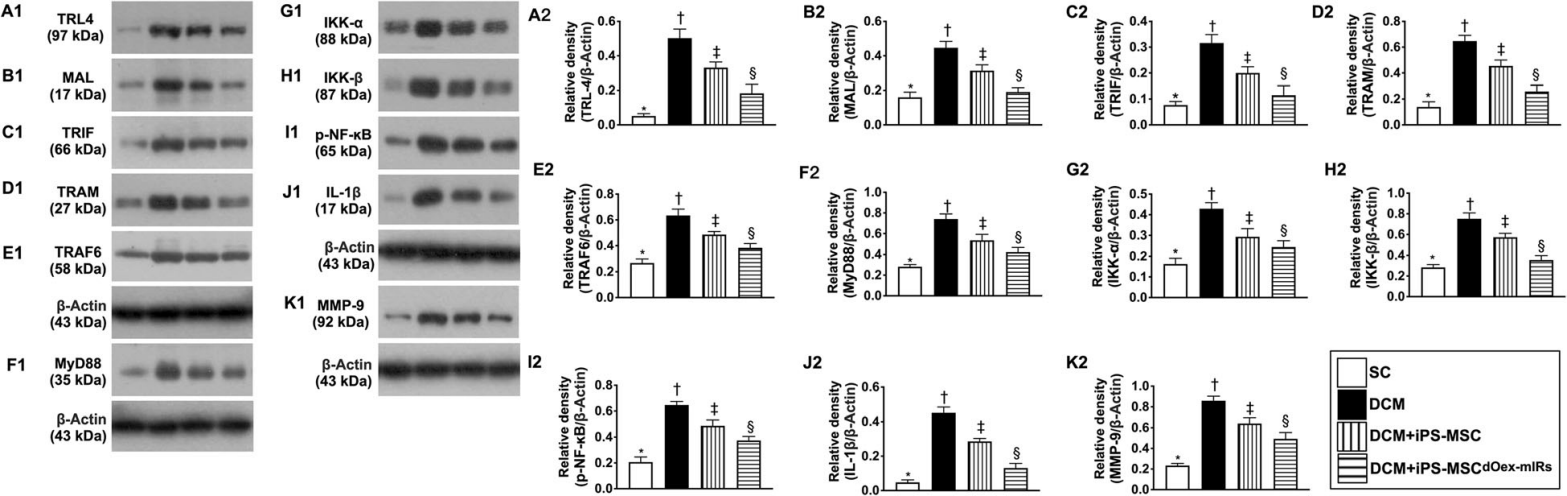
Protein expressions of upstream and downstream inflammatory signaling pathways in LV myocardium by day 60 after DCM induction. **A1**, **A2** Protein expression of toll-like receptor 4 (TLR4), * vs. other groups with different symbols (†, ‡, §), *p*< 0.0001. **B1**, **B2** MyD88 adaptor-like (MAL), * vs. other groups with different symbols (†, ‡, §), *p*< 0.0001. **C1**, **C2** Protein expression of Toll/IL-1R domain-containing adaptor-inducing IFN-β (TRIF), * vs. other groups with different symbols (†, ‡, §), *p*< 0.0001. **D1**, **D2** Translocating chain-associated membrane protein (TRAM), * vs. other groups with different symbols (†, ‡, §), *p*< 0.0001. **E1**, **E2** Protein expression of TNF receptor-associated factor 6 (TRAF6), * vs. other groups with different symbols (†, ‡, §), *p*< 0.0001. **F1**, **F2** Protein expression of myeloid differentiation primary response 88 (MyD88), * vs. other groups with different symbols (†, ‡, §), *p*< 0.0001. **G1**, **G2** Protein expression of IκB Kinase α (IKK-α), * vs. other groups with different symbols (†, ‡, §), *p*< 0.0001. **H1**, **H2** Protein expression of IKK-ß, * vs. other groups with different symbols (†, ‡, §), *p*< 0.0001. **I1**, **I2** Protein expression of phosphorylated nuclear factor ß (p-NF-κß), * vs. other groups with different symbols (†, ‡, §), *p*< 0.0001. **J1**, **J2** Protein expressions of interleukin (IL)-1ß, * vs. other groups with different symbols (†, ‡, §), *p*< 0.0001. **K1**, **K2** Protein expression of matrix metalloproteinase (MMP)-9, * vs. other groups with different symbols (†, ‡, §), *p*< 0.0001. All statistical analyses were performed by one-way ANOVA, followed by Bonferroni multiple comparison post hoc test (*n*=6 for each group). Symbols (*, †, ‡, §) indicate significance (at 0.05 level). LVEF = left ventricular ejection fraction; DCM = dilated cardiomyopathy; iPS-MSCs = inducible pluripotent stem cell-derived mesenchymal stem cells; dOex-mIRs = double overexpression of microRNAs

**Fig. 11:**
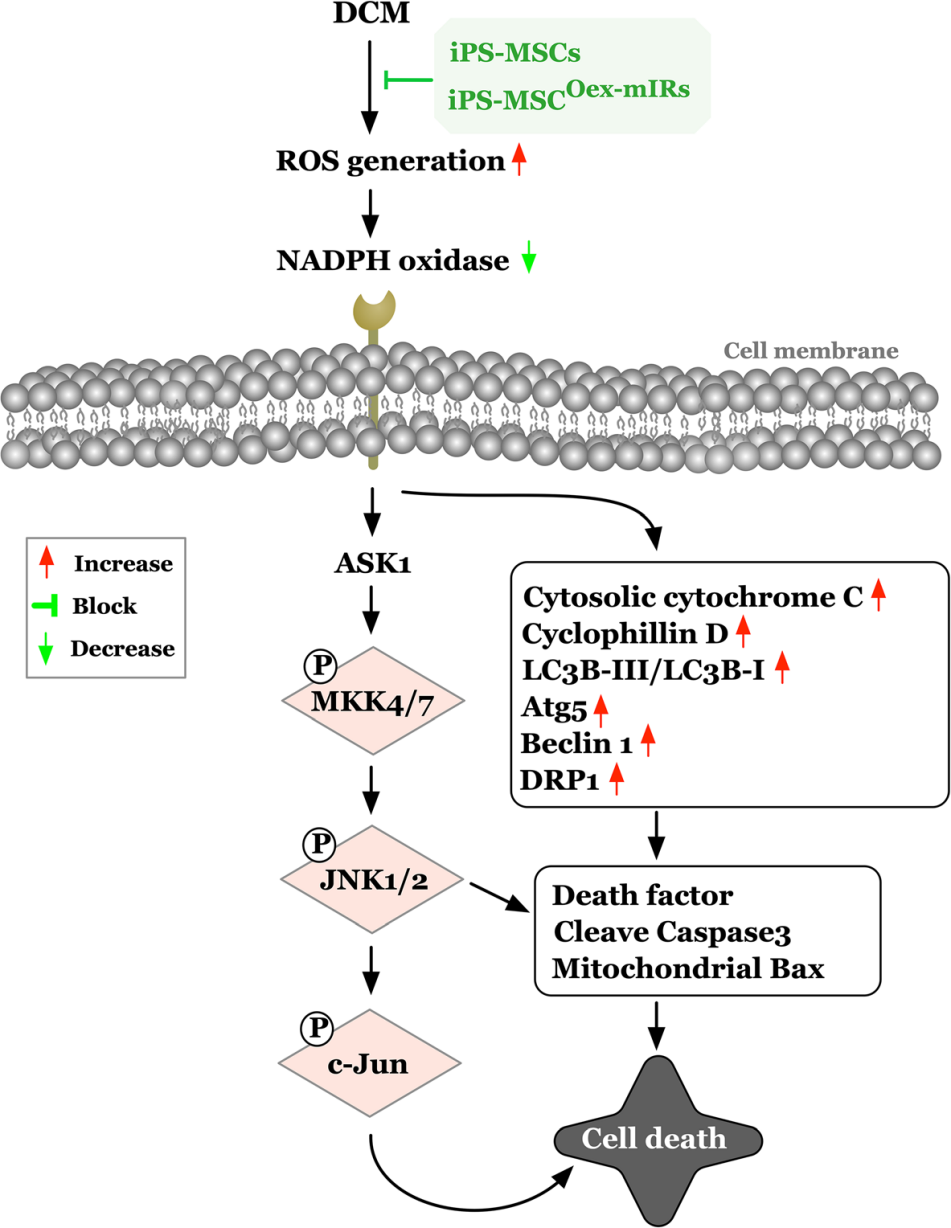
Schematically illustrate the upstream and downstream inflammatory signaling pathways on DCM rodent. DCM = dilated cardiomyopathy; iPS-MSCs = inducible pluripotent stem cell-derived mesenchymal stem cells; dOex-mIRs = double overexpression of microRNAs

To elucidate the inflammatory signalings in the LV myocardium in the DCM setting, the Western blot was utilized. The result showed that the protein expressions of TLR4, MyD88, MAL, TRIF, TRAM, TRAF6, IKKα, IKKß, and p-NF-κß, nine indicators of upstream signaling, and protein expressions of TNF-α, IL-1ß, and MMP-9, three indices of downstream signaling, were lowest in group 1, highest in group 2, and significantly lower in group 4 than in group 3 (Fig. 10). Our findings clearly highlighted the upstream and downstream inflammatory signalings involved in the initiation and propagation of myocardial damage in DCM animals (refer to Fig. 11).

## Discussion

This study which investigated the therapeutic impact of dOex-mIRs of iPS-MSCs on protecting the heart against DCM damage yielded several striking implications. First, rather than only single mechanism, this study identified that the underlying mechanisms of DCM caused heart and myocardium dysfunction were quite complicated (refer to Figs. 8 and 11). Second, as compared with the SC group, the LVEF was significantly progressively worsening in DCM only, suggesting our DCM model in rodent was successfully created for the study. Third, the LVEF was significantly preserved by iPS-MSCs and further significantly preserved by iPS-MSC^dOex-mIRs^ in DCM rodent, highlighting that this therapeutic management may be potential in the future for DCM patients, especially when their decompensated HF is refractory to conventional therapy that demands the final resort of heart transplantation.

It is well recognized that no matter how advanced pharmaceutical and accessorily mechanical devices have been utilized for those DCM patients with end-stage decompensated HF and poor LV function, the therapeutic success is still extremely limited, resulting in an unacceptably high annual mortality in these patients. Accordingly, heart transplantation, a conventional therapy, could serve as the last resort for these patients. However, the donor of a living heart is extremely lacking, prompting scientists to seek an alternative modality with safety and efficacy. Intriguingly, growing data have demonstrated that cell therapy effectively improved ischemia-related organ dysfunction through tissue regeneration, angiogenesis, anti-inflammation, and oxidative stress as well as immunomodulation [30, 34–36, 38, 40, 41]. One important finding in the present study was that as compared with DCM animals, the LVEF (i.e., the heart function) was significantly preserved in iPS-MSCs treated DCM animals. Our finding corroborated with the finding of the previous studies [30, 34–36, 38, 40, 41]. The most important finding in the present study was that iPS-MSC^dOex-mIRs^ was expected superior to iPS-MSCs for improving the LVEF in DCM rodent. As we expected, our finding, in addition to the extension of the previous studies, highlights that this strategic management may pose a therapeutic potential for those DCM patients with decompensated HF and poorest heart function with requirement of heart transplantation.

It is always a universal concept that prior to offering an effective treatment for a specific disease, the delineation of the underlying mechanism of the disease entity is of utmost importance. An important finding in the present study was that the signaling pathway of oxidative stress, mitochondrial damaged ,and apoptotic biomarkers as well as the downstream members of the MAPK family were identified to be markedly enhanced in DCM animals (refer to Fig. 11). Of particular importance was that not only the in vitro but also the in vivo studies demonstrated that the upregulated oxidative-stress signaling further elicited the mitochondrial damage, apoptosis, and autophagic activity in iPS-MSC treated by H_2_O_2_ and DCM myocardium. Intriguingly, previous studies have also clearly identified that these aforementioned molecular-cellular perturbations were remarkably enhanced in the DCM setting [29, 42] and cardiorenal syndrome [43, 44] and those of MAPK family members in myocardial ischemia [45]. Accordingly, the findings of the in vitro and in vivo studies, in addition to being consistent with the findings of the previous studies [29, 42–44], could, at least in part, explain why the LVEF was substantially reduced in DCM animals than in those of SC animals.

Abundant data have revealed that inflammatory activation was frequently elicited in DCM myocardium [29, 42], acute myocardial infarction [45], and cardiorenal syndrome [43, 44], which in turn led to progressively cardiomyocyte apoptosis and death, resulting in myocardial fibrosis and deteriorating heart function [29, 42–45]. A principal finding in the present study was that not only the upstream but also the downstream inflammatory signalings in the DCM setting were clearly delineated (refer to Fig. 8). In this way, our findings, in addition to strengthening the findings of previous studies [29, 42–45], further identified that the underlying signaling pathway of DCM was complex and probably involved in multiple signaling pathways (i.e., inflammation, oxidative stress, MAPK family, and autophagy) (refer to Figs. 8 and 11). Of particularly distinctive finding was that iPS-MSC^dOex-mIRs^ treatment was superior to iPS-MSCs treatment for improving LVEF in DCM animals.

Perhaps, the readers would be interesting not only in the exactly underlying mechanisms of DCM but much more interesting in the mechanistic basis of how iPS-MSCs and iPS-MSC^dOex-mIRs^ treatment on improving the rat heart function. In our schematically proposed mechanisms of Figs. 8 and 11, we clearly delineated that the iPS-MSCs and iPS-MSC^dOex-mIRs^ treatment on successful preservation of the heart function was mainly through suppressing the upstream and downstream inflammatory, cell-stress and oxidative-stress signalings to avoid the mitochondrial damage, cell apoptosis, DNA damage, and myocardial fibrosis in DCM rodent.

### Study limitation

This study has limitations. First, although the study period was 60 days, the longer-term impact of iPS-MSCs/ iPS-MSC^dOex-mIRs^ on preservation of the left ventricular function is still currently uncertain. Second, in the absence of applying 2^nd^ iPS-MSC^dOex-mIRs^, whether a 2nd therapy would offer additional benefit on furthermore improving the cardiac function in those DCM animals remains to be answered.

In conclusion, as compared to the iPS-MSCs therapy iPS-MSC^dOex-mIRs^ therapy offered additional benefits on improving the LVEF in DCM animals.

## Conclusion

iPS-MSC^dOex-mIRs^ therapy was superior to iPS-MSC therapy for preserving LV function in DCM rat.

## Supplementary Information

**Additional file 1: Figure S1.** Schematically illustrate the step-by-step procedure of cell culturing for the iPS derived into iPS-MSCs.

**Additional file 2: Figure S2.** Illustrating the time courses of differentiation of iPS to iPS-MSCs. iPS = inducible pluripotent stem cell; iPS-MSCs = inducible pluripotent stem cell derived-mesenchymal stem cells.

**Additional file 3: Figure S3.** Illustrating the iPS-MSC differentiated into adipocytes, chondrocytes, osteoblast. A to C) Illustrating the adipogenic differentiation of iPS-MSCs into adipocytes stained by Oil red O. D to F) Illustrating the chondrogenic differentiation of iPS-MSCs into chondrocytes stained by Alcian Blue. G to I) Illustrating the osteogenic differentiation of iPS-MSCs into osteoblast stained by Alizarin Red S.

## Data Availability

The data that support the findings of this study are available from the corresponding authors upon reasonable request.

## Abbreviations

dOex-mIRs: Double overexpression of miR-19a and miR-20a
iPS: Human induced pluripotent stem cell
MSCs: Mesenchymal stem cells
LVEF: Left ventricular ejection fraction
DCM: Dilated cardiomyopathy
HF: Heart failure
ROS: Reactive oxygen species
iPSC-MSCs: Human induced pluripotent stem cell-derived mesenchymal stem cells
CKD: Chronic kidney disease
Dox: Doxorubicin
SD: Sprague-Dawley
IP: Intraperitoneal
2D: Two-dimensional
LV: Left ventricular
ESD: End-systolic diameter
EDD: End-diastolic diameter
PVDF: Polyvinylidene difiuoride
TNF: Tumor necrosis factor
NF: Nuclear factor
TRAF6: Tumor necrosis factor receptor-associated factor 6
TLR: Toll-like receptor
MyD88: Myeloid differentiation primary response 88
Mal: Myelin and lymphocyte protein
TRAM: Translocating chain-associated membrane protein
TRAF6: TNF receptor associated factor 6
IKB-α: Nuclear factor of kappa light polypeptide gene enhancer in B cell inhibitor, alpha
ASK1: Apoptosis signal-regulating kinase 1
p-MMK4: Phosphorylated mitogen-activated protein kinase 4
IL: Interleukin
MMP: Matrix metalloproteinase
DRP1: Dynamin-related protein 1
c-PARP: Cleaved Poly (ADP-ribose) polymerase
TGF: Transforming growth factor
HRP: Horseradish peroxidase
ECL: Enhanced chemiluminescence
IHC: Immunohistochemical
IF: Immunofluorescent
8-OHdG: 8-hydroxy-2′-deoxyguanosine
HPFs: High-power fields
ROS: Reactive oxygen species
IFI: Increased fluorescence intensity
BFI: Baseline fluorescence intensity
MAPK: Mitogen-activated protein kinase
