# Supplementary figures and images for: Double overexpression of miR-19a and miR-20a in induced pluripotent stem cell-derived mesenchymal stem cells effectively preserves the left ventricular function in dilated cardiomyopathic rat
Source: Stem Cell Res Ther. 2021 Jun 29;12:371. doi: 10.1186/s13287-021-02440-4 (PMC8243466; doi:10.1186/s13287-021-02440-4)

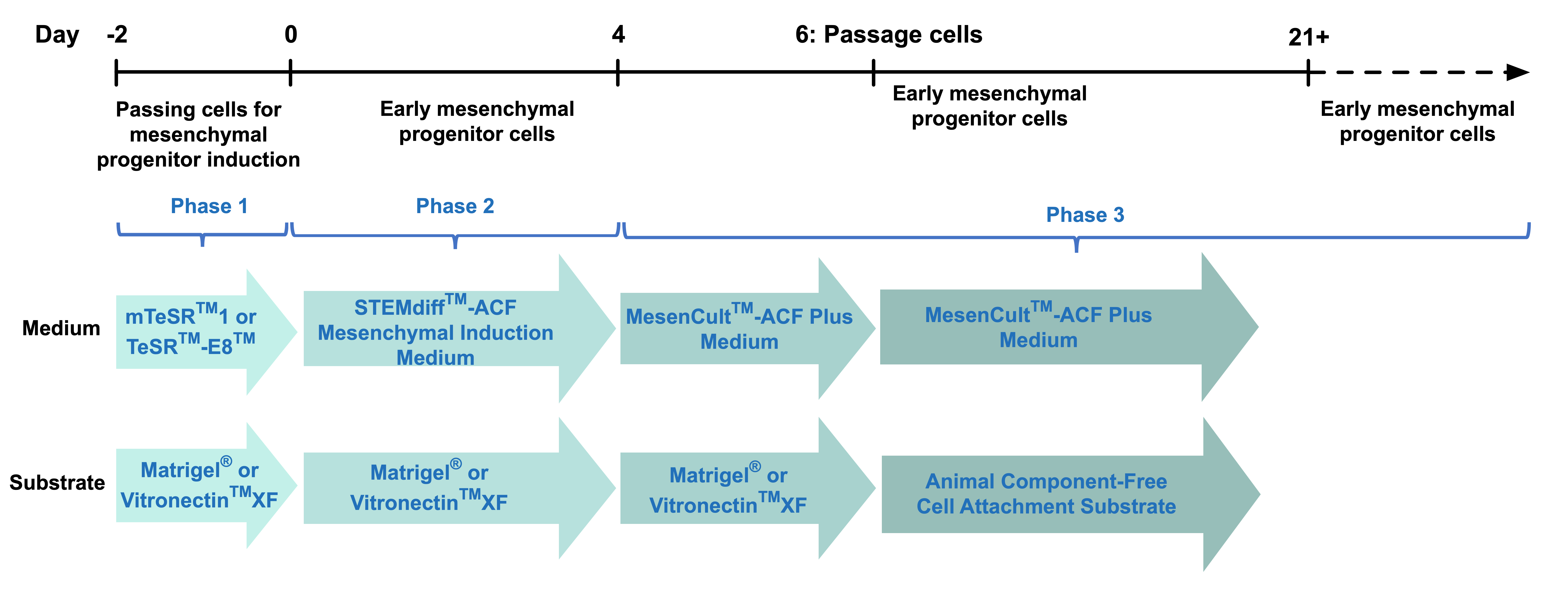

Supplement: Supplementary file 1 — Additional file 1: Figure S1. Schematically illustrate the step-by-step procedure of cell culturing for the iPS derived into iPS-MSCs. [file 13287_2021_2440_MOESM1_ESM.jpg]

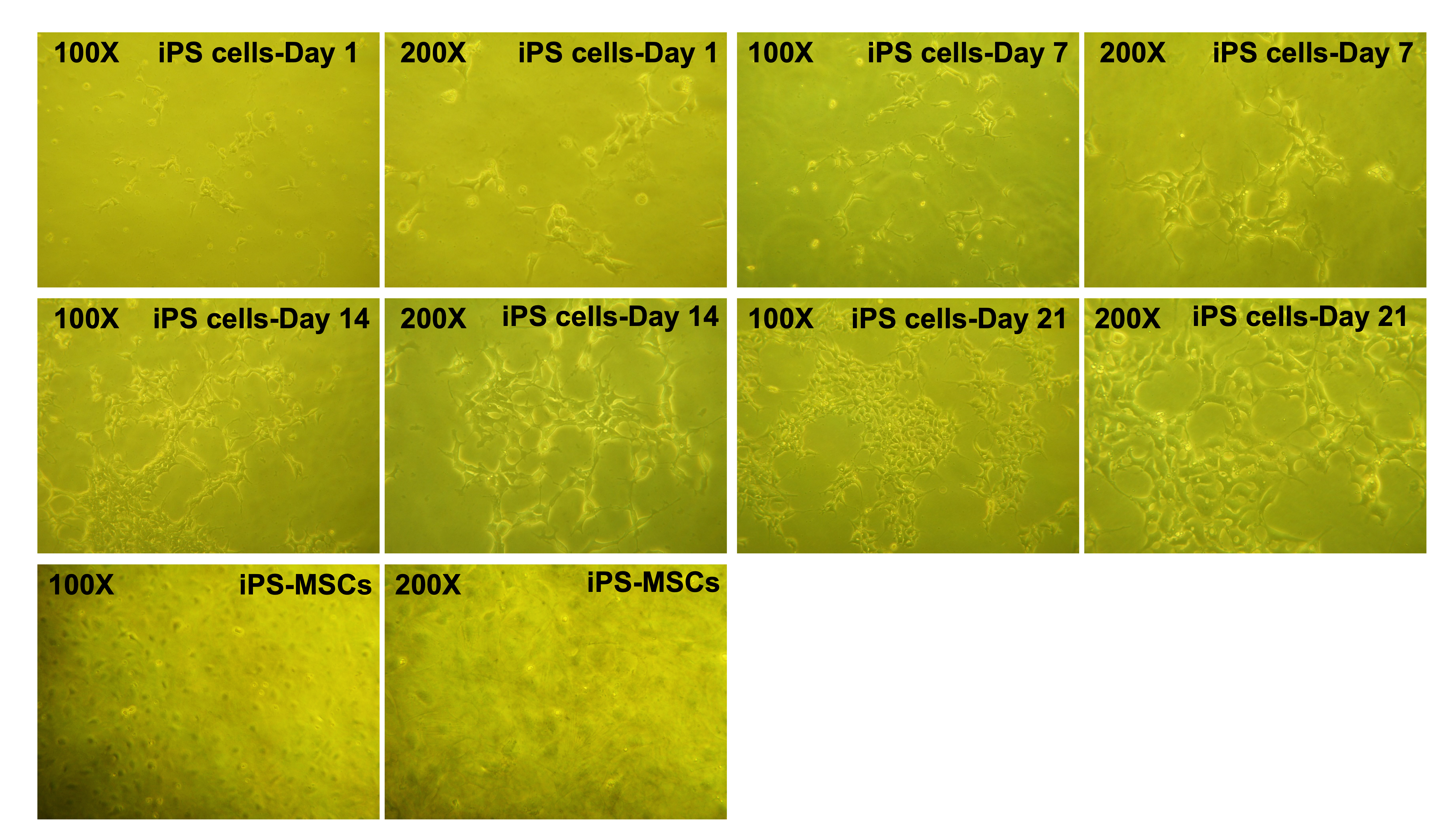

Supplement: Supplementary file 2 — Additional file 2: Figure S2. Illustrating the time courses of differentiation of iPS to iPS-MSCs. iPS = inducible pluripotent stem cell; iPS-MSCs = inducible pluripotent stem cell derived-mesenchymal stem cells. [file 13287_2021_2440_MOESM2_ESM.jpg]

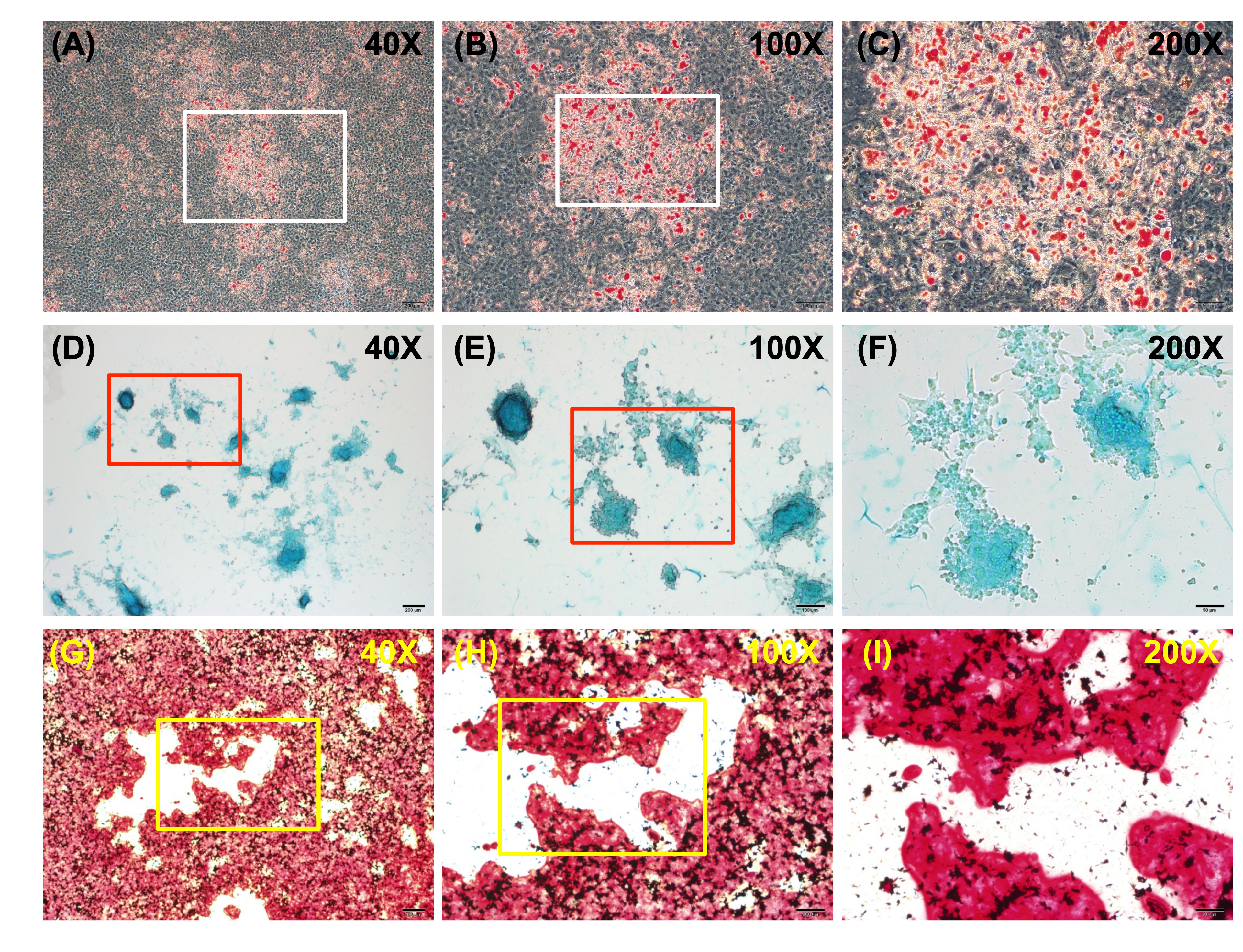

Supplement: Supplementary file 3 — Additional file 3: Figure S3. Illustrating the iPS-MSC differentiated into adipocytes, chondrocytes, osteoblast. A to C) Illustrating the adipogenic differentiation of iPS-MSCs into adipocytes stained by Oil red O. D to F) Illustrating the chondrogenic differentiation of iPS-MSCs into chondrocytes stained by Alcian Blue. G to I) Illustrating the osteogenic differentiation of iPS-MSCs into osteoblast stained by Alizarin Red S. [file 13287_2021_2440_MOESM3_ESM.jpg]
